# Management and Novel Treatment of Degloving Soft Tissue Injuries: A Case Report

**DOI:** 10.7759/cureus.49999

**Published:** 2023-12-05

**Authors:** Ali A Raza, Monica J Whaley, Murtaza Shakir

**Affiliations:** 1 Department of Research, Alabama College of Osteopathic Medicine, Dothan, USA; 2 Department of General Surgery, Memorial Hermann Health Systems, Houston, USA

**Keywords:** general trauma surgery, amniotic stem cells, motor vehicle accident, general and vascular surgery, degloving wounds

## Abstract

Degloving soft tissue injuries (DSTIs) are injuries usually due to trauma sustained parallel to the body, shearing off skin and soft tissue from the underlying muscle and bone. Most commonly due to workplace and traffic accidents, DSTIs can occur due to a variety of traumas, resulting in a wide spectrum of clinical presentations. Treatment is often prolonged and complex, which requires the intervention of a multidisciplinary healthcare team to provide the most optimal outcomes. In this case, we report an 89-year-old male presenting with a lower left leg degloving injury due to a single motor vehicle accident (MVA). Initial surgery and observation were performed to determine the vascular structure, and subsequent debridement was performed to assess the viability of the tissue. Aggressive weekly debridement with stem cell injections and membrane wraps were used to stimulate healing in the patient. Once completed, a skin flap was grafted, which led to the patient completing his healthcare plan within six months. This case serves as an example of an effective healthcare team providing unified care to their patients to increase their mobility and quality of life.

## Introduction

Degloving soft tissue injuries (DSTIs) represent complex and often devastating traumas that pose significant challenges to both patients and healthcare providers alike. These injuries occur when a substantial portion of the skin and subcutaneous tissue is forcibly separated from the underlying fascia and muscles [1]. Understanding the nature of degloving injuries, distinguishing between open and closed variants, determining the mechanism behind these injuries, and being aware of current treatment guidelines are requisites for optimizing patient outcomes. 

One critical distinction in the realm of degloving injuries is the differentiation between open and closed cases. Open degloving injuries involve direct exposure of the wound to the external environment, often resulting from severe trauma, such as motor vehicle accidents (MVAs) or heavy machinery injuries. Conversely, closed degloving injuries manifest without an open wound and typically occur due to shearing forces or crushing injuries. Recognizing the difference between presentations is paramount as it significantly impacts treatment strategies [2].

DSTIs may encompass a wide variety of traumatic forces, each presenting with a relatively unique challenge in terms of tissue damage and subsequent management. High-energy traumas, usually stemming from MVAs and industrial accidents, can subject the body to extremely high-impact forces, resulting in DSTIs that may span large body regions and may have additional fractures or internal injuries [3]. Crush injuries, also seen in industrial accidents, or falls, generate forces that separate skin and soft tissue from underlying structures [1]. Shearing forces sustained during MVAs or bicycle accidents may come from the skin being propelled against fixed objects. Regardless of the mechanism of injury, DSTIs are infamous for their potential to inflict significant damage to the skin and beneath the surface [4].

To effectively manage a DSTI, a multidisciplinary approach is essential, involving general surgeons, trauma specialists, and wound care experts. Early evaluation and stabilization of the patient's overall condition are required, with immediate attention to life-threatening injuries and vitals such as airway, breathing, and circulation. Surgical debridement plays a pivotal role, facilitating the removal of necrotic tissue, reducing infection risks, and accurately assessing the extent of soft tissue loss [5]. Depending on the severity and location of the degloving injury, various reconstructive techniques, including skin grafts, local flaps, or stem cell injections, may be employed [1]. Additionally, meticulous wound care, antibiotic prophylaxis, and strict adherence to sterile techniques are important for infection prevention [6]. Finally, functional rehabilitation through physical and occupational therapy is crucial to restore function and enhance the patient's quality of life.

In this case report, we present an account of an 89 year-old male presenting with a DSTI occurring after a single-vehicle accident. We hope to shed light on the mechanisms of injury involved and to demonstrate novel treatments that empowered this patient to achieve a successful recovery. By discussing the success of a multidisciplinary approach, we aim to contribute to the growing body of knowledge surrounding DSTIs and ultimately enhance patient outcomes when facing this immense challenge.

## Case presentation

We present the case of an 89-year-old male who was brought to the emergency department (ED) following a single MVA. The patient exited his vehicle to open his house gate and mistakenly put the car in reverse. He then ran after the car to attempt to gain control, but was knocked over by the front door, with his right lower extremity (RLE) being run over by the vehicle. The patient denies hitting his head, loss of consciousness, or any other injury other than the injury to the RLE. He was taken by emergency medical services (EMS) to the local ED, where he was then evaluated. 

The patient has a significant medical history including hypertension, hyperlipidemia, and type 2 diabetes mellitus that are well controlled with medication prescribed and managed by his Family Medicine physician. He denies any history of cognitive impairment or falls due to gait instability. The patient is able to ambulate at home without assistance. He had a cholecystectomy 12 years ago with no complications and has never been hospitalized. He also denies any tobacco use, alcohol use, or any illicit drug use.

**Figure 1:**
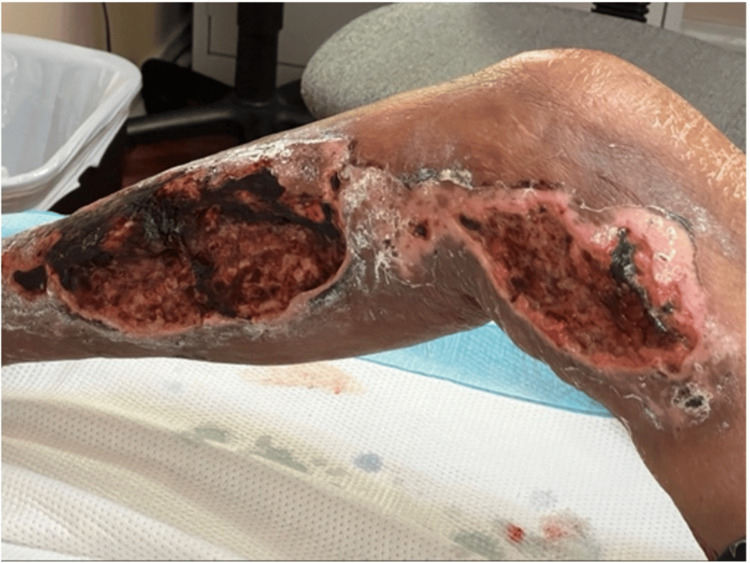
Initial presentation of degloving soft tissue injury

The patient presented to the local ED via EMS with complaints of RLE pain due to an injury that occurred in a single MVA two hours prior (Figure 1). At the time of the accident, the family noted that the patient was not disoriented and was alert throughout. EMS was called due to the patient's advanced age.

On initial exam, the patient's vitals included a heart rate of 96 beats per minute, a blood pressure of 158/92, a respiratory rate of 20 breaths per minute, a temperature of 98.8°F, and an oxygen saturation of 97% on room air. The patient was awake, alert, oriented, and in moderate amounts of pain due to RLE injury. Cardiovascular exam revealed tachycardia, regular rhythm, and no murmurs, gallops, or thrills. Pulmonary exam revealed no increased work of breathing, and the lungs sound clear to auscultation bilaterally. Musculoskeletal exam was significant for RLE laceration that was tender to palpation and restricted right hip range of motion due to pain. CT scans of the head, C-spine, chest, and abdomen were performed and showed no significant injuries. X-rays of the pelvis and lower legs were obtained that confirmed no fractures throughout.

The patient was treated with intravenous analgesics to achieve pain control while in the ED. A surgery consult was obtained, and the patient was taken to the operative room for washout and debridement. Once in the operative room, RLE was prepped and draped in the usual sterile fashion. Extensive washout was initially performed, with subsequent debridement. All bleeding points were coagulated with electrocautery. The wound measured 15 cm x 10 cm and was then covered in extensive amounts of Xeroform (Cardinal Health, Dublin, Ohio, United States). The patient tolerated the procedure well and with no complications. 

**Figure 2:**
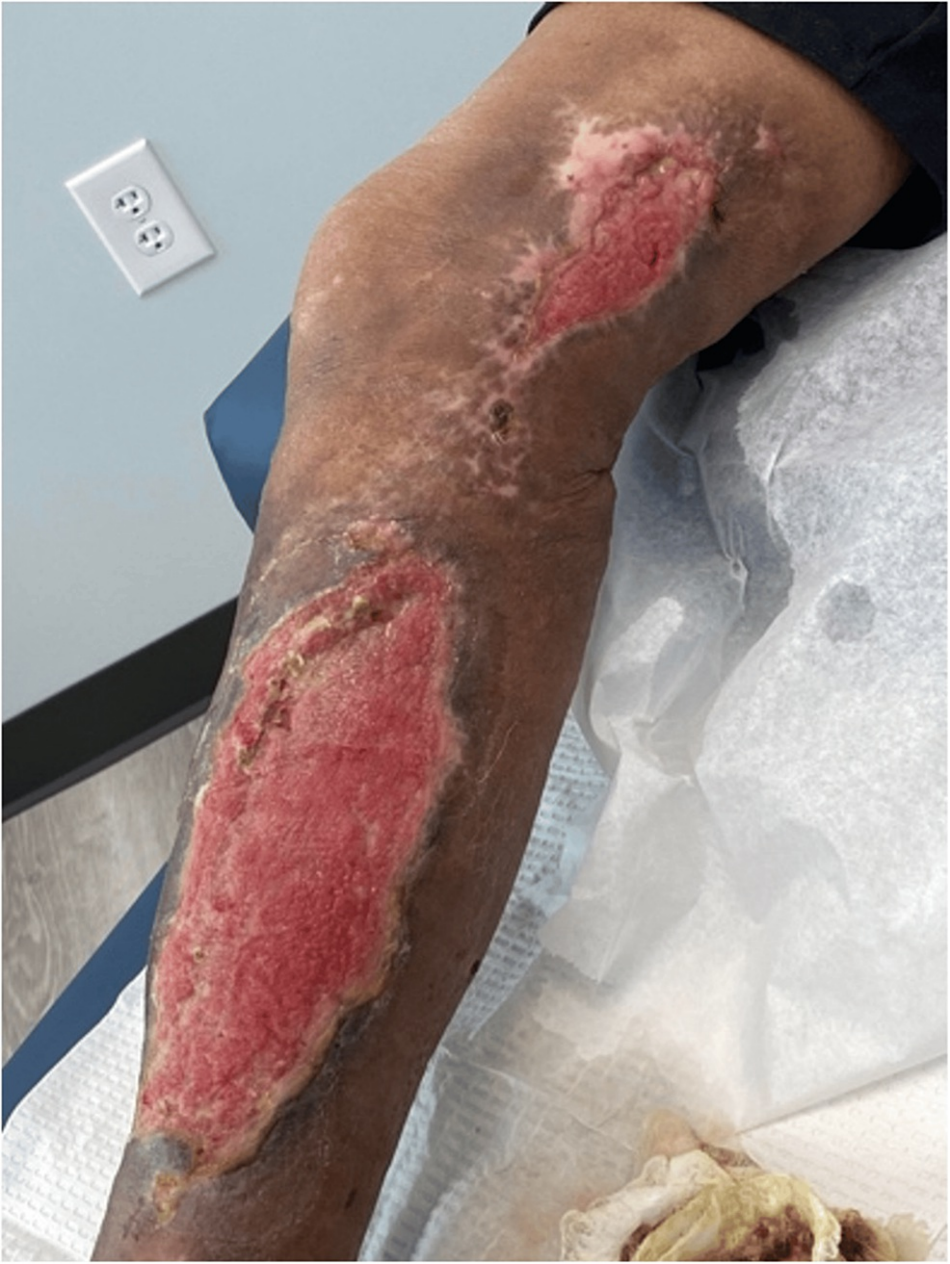
Injury at six weeks' recovery

**Figure 3:**
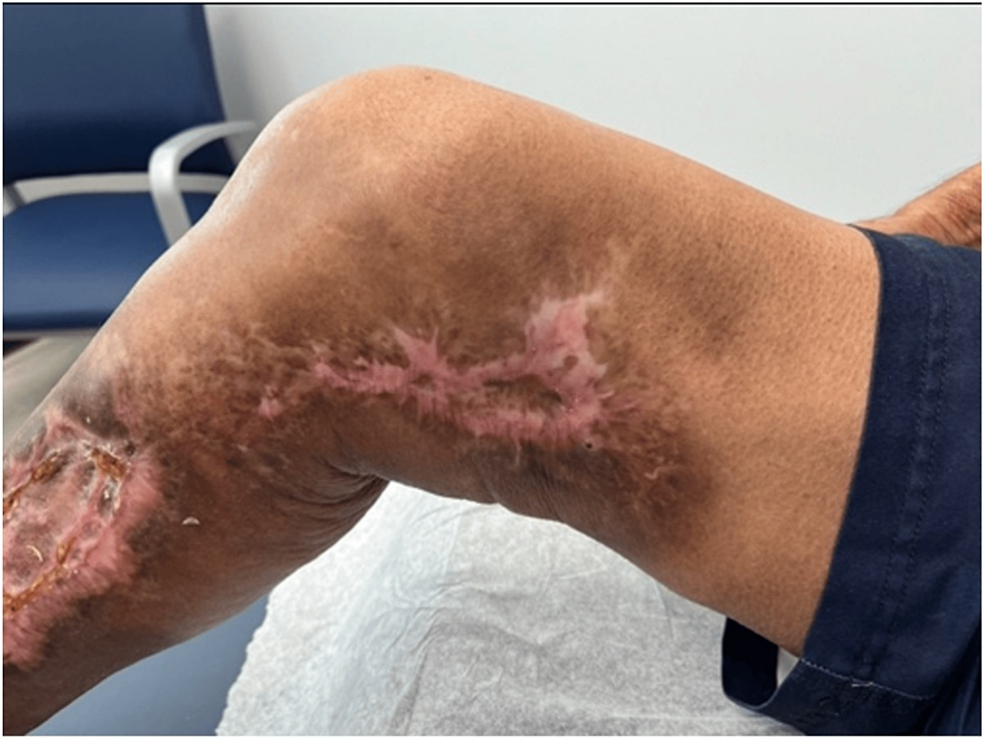
Injury at six months' recovery

The patient was discharged several days later and followed up in surgery office twice a week for the next eight weeks for further debridement, dressing changes, and amniotic stem cell injections. He tolerated these appointments well, and six weeks after the initial injury, he started physical therapy (PT) (Figure 2). While in PT, the patient regained most of his mobility and function with the use of a cane. At the conclusion of regular appointments, he underwent a skin graft to cover any remaining exposed wounds. The patient tolerated the skin graft surgery well with no complications and no further procedures required. He continued with PT for six months after the skin graft (Figure 3).

## Discussion

DSTIs are traumatic injuries that can severely impact one's quality of life if not managed and treated quickly. Usually those who have sustained a traumatic injury like a DSTI present acutely to the ED, where the healthcare team then will pursue the best course of action. This case highlights the successful management of a challenging DSTI, a complex clinical scenario, particularly in the elderly population. A degloving injury results from the separation of the skin and subcutaneous tissues from the underlying fascia and muscle, which can lead to extensive soft tissue damage. This type of injury often presents with complications due to its potential to compromise blood supply, increase the risk of infection, and significantly delay wound healing [7].

In this case, we employed a novel approach using amniotic liquid allografts to promote wound healing, and the patient demonstrated remarkable progress throughout the course of treatment. Preservation of skin tissue and subsequent skin grafting is very important in the treatment of these patients [8], but due to the variable clinical presentation, there is no single line of treatment that is conducted with every patient undergoing a degloving treatment [9]. However, a general guideline of debridement and closure is generally followed. In this clinical case, a combination of debridement, fluid growth factor (FGF) (which is injected as stem cells), membrane wraps, compression wraps, and skin flap reconstruction was used [10]. While most of these techniques have been implemented routinely, the literature surrounding FGF and amniotic stem cells is lacking [11]. This report serves to provide a case in which these techniques were relied upon and the patient had a successful outcome.

The utilization of amniotic liquid allografts, such as FGF, in this treatment of a DSTI has shown promising results. These allografts contain a variety of growth factors, cytokines, and extracellular matrix components that can enhance the regenerative capacity of damaged tissues. According to a protein analysis conducted by BioLabs [12], there are several proteins that carry out several cellular processes, including cellular growth and differentiation, angiogenesis, and tissue remodeling, and have anti-inflammatory/anti-microbial activity, all of which aid in the healing of various types of wounds, including soft tissue injuries. Similarly, an alternative treatment method involved amniotic allograft membranes, which also serves to stimulate several different cellular processes. The membrane is a fibrous scaffold that allows for the repair, reconstruction, replacement, or supplementation of a tissue. Although it can be used for several types of wounds, it is efficacious for those that present with larger acute wounds.

Elderly patients often present unique challenges when managing traumatic injuries. Advanced age is frequently accompanied by comorbidities, frailty, and diminished physiological reserve, all of which can complicate wound healing and increase the risk of complications. In our patient, his age and comorbidities added complexity to this case [13]. His self-care was limited, but the family was able to assist with his postoperative care and rehabilitation. This underscores the importance of individualized care plans in conjunction with a reliable support system [14].

The significance of a multidisciplinary team and a support system in managing complex cases like this cannot be overstated. Elderly patients with traumatic injuries often require a comprehensive approach that addresses both their physical and psychosocial needs [15]. Such a team can ensure that all aspects of care are considered, including the prevention and management of complications, optimizing functional outcomes, and addressing patient and family concerns.

## Conclusions

The successful management of a DSTI in an elderly patient using amniotic liquid allografts underscores the potential of regenerative medicine in wound healing. This case also highlights the importance of collaboration from a multidisciplinary team and how we can best address the diverse and complex needs of elderly patients and their family. This collaborative model can enhance patient outcomes and quality of life. Further research is needed to refine best practices for managing such acute injuries in elderly individuals.
